# Practice Patterns in Body Mass Index Optimization Among US Arthroplasty Surgeons: Results of a National American Association of Hip and Knee Surgeons Survey

**DOI:** 10.5435/JAAOSGlobal-D-25-00187

**Published:** 2026-02-17

**Authors:** Timur Seckin, Paul Tesoriero, Samuel Zverev, Philip Spadafora, Chelsea Sicat, Gregory Sirounian, Jan Albert Koenig

**Affiliations:** From the Burnett School of Medicine, Texas Christian University, Fort Worth, TX (Mr. Seckin); the NYU Langone Long Island Hospital, Mineola, NY (Mr. Seckin, Mr. Zverev, Mr. Spadafora, Dr. Sicat, and Dr. Sirounian); the 1st Year Upper Extremity and Hand Fellowship, Columbia University, New York, NY (Dr. Tesoriero); and the Department of Orthopedic Surgery, NYU Langone Long Island Hospital, Mineola, NY (Dr. Koenig).

## Abstract

**Introduction:**

As total hip arthroplasty and total knee arthroplasty procedures are increasingly performed on younger patients with obesity, the optimal approach to preoperative weight management remains undefined. This study explores national trends among US arthroplasty surgeons regarding body mass index (BMI) cutoffs, weight optimization strategies, and weight loss medication usage.

**Methods:**

A 28-question national survey was distributed through e-mail to members of the American Association of Hip and Knee Surgeons and shared on professional social media platforms (Facebook, ResearchGate, and LinkedIn) between January 1 and August 5, 2024.

**Results:**

Regarding arthroplasty reconstruction fellowship training, 82.58% (441/534) of respondents were fellowship trained, 11.42% (61/534) were not fellowship trained, and 5.99% (32/534) trained in other specialties. In terms of BMI optimization strategies, 82.84% (n = 444/536) recommended structured diet and exercise programs, 77.99% (n = 418/536) recommended dietitian or weight loss specialist programs, and 52.61% (n = 282/536) advocated for bariatric surgery. For total hip arthroplasty, 45.13% of surgeons used a BMI cutoff <40 kg/m^2^ (n = 241/534), followed by 23.97% advocating for no strict cutoff (n = 128/534). For total knee arthroplasty, 41.65% reported using a cutoff of <40 kg/m^2^ (n = 222/533), with 24.39% (n = 130/533) reporting no strict BMI cutoff. In addition, 27.62% (n = 58/210) reported that their patients used weight loss medications such as GLP-1 agonists. Notably, 68.0% (n = 356/526) of surgeons allowed up to 1 to 2 years for BMI optimization before surgery.

**Conclusion:**

Although many arthroplasty surgeons use BMI cutoffs, many accommodate patients through nonsurgical interventions to facilitate weight loss. These findings indicate that many respondents report a multidisciplinary approach to preoperative BMI optimization.

From 2021 to 2023, the Centers for Disease Control and Prevention reported that the prevalence of obesity, defined as a body mass index (BMI) of 30 kg/m^2^ or higher, was 40.3% among American adults.^1^ Obesity has been associated with an increased risk of developing osteoarthritis in both the hip and the knee.^2,3^ Patients undergoing total hip arthroplasty (THA) and total knee arthroplasty (TKA) are increasingly younger and more likely to have obesity.^4^ By the year 2029, the predicted prevalence of obesity or morbid obesity in primary TKA and THA patients is projected to be 69% and 55%, respectively.^5,6^

In patients with obesity undergoing TKA or THA, obesity is associated with higher rates of revision surgery, implant revision or removal, aseptic loosening, and deep periprosthetic infection.^7-9^ Subsequently, arthroplasty surgeons have used BMI measurements and employed cutoffs as preoperative screening tools.^10,11^ However, the use of strict BMI cutoffs has also been associated with decreased eligibility and access to arthroplasty for Black, Hispanic, and female patients.^12^ BMI optimization, defined as the intentional reduction of excess body weight before surgery, serves as a targeted preoperative strategy to improve candidacy for total joint arthroplasty.

BMI optimization includes nutritional consultations, physical therapy–assisted exercise programs, the use of weight loss–promoting medications, and bariatric surgical interventions to facilitate BMI reductions before surgery. A systematic review (n = 7 studies) demonstrated that nonsurgical weight loss interventions resulted in a 5.0 to 32.5 kg reduction in weight and a 3 to 12 kg/m^2^ BMI reduction for preoperative elective TKA patients with obesity.^13^ Although bariatric surgery has shown mixed results in reducing postoperative risk,^14,15^ comparative studies demonstrate that patients undergoing Total Joint Arthoplasty (TJA) typically achieve a 14 kg/m^2^ reduction in BMI.^14,16^ A retrospective cohort study analyzed the use of Glucagon-like Peptide-1 (GLP-1) receptor agonists in 268,504 THA and 386,356 TKA patients, finding that preoperative GLP-1 use (n = 1044 for THA; n = 2095 for TKA) was associated with a 42% decreased risk of periprosthetic joint infection (PJI) and a 47% decreased risk of readmission, respectively, within 90 days postoperation, with no mention of weight loss outcomes.^17^

Despite the growing prevalence of obesity and its well-documented effect on arthroplasty outcomes, there is a paucity of data regarding arthroplasty surgeons preoperative weight management. Specifically, there is limited data on the preferred use of BMI thresholds, nonsurgical weight loss interventions, and emerging pharmacologic therapies. This study aims to define current practices and decision-making frameworks among US. arthroplasty surgeons, with the goal of guiding standardized, evidence-based optimization protocols for this high-risk patient population.

## Methods

A voluntary online questionnaire-based survey was distributed to the American Association of Hip and Knee Surgeons (AAHKS) registered surgeons through e-mail. The 28 questions (Online Appendix 1, http://links.lww.com/JG9/A496) were developed by a multidisciplinary team, including arthroplasty surgeons, bariatric surgery fellows, and orthopaedic surgery residents, to address existing practices in BMI optimization and challenges in managing patients with obesity undergoing TJA. The questionnaire included multiple-choice questions regarding the type of practice, fellowship training, surgical approaches for patients with obesity, use of technology, BMI cutoffs for surgery, recommendations for BMI optimization, follow-up schedules, and specific perioperative and postoperative protocols for patients with elevated BMI. The survey was anonymous; no direct identifiers or geographic location were collected. Data reflect self-reported practices at a single time point. Given its cross-sectional, self-reported design, this study represents Level V evidence.

The survey started on January 1, 2024, and closed for analysis on August 5, 2024. It was distributed twice through e-mail to AAHKS members and shared on professional social media platforms (Facebook, ResearchGate, and LinkedIn). Questions 25 to 28—focusing on the use, route of administration, management, and timing of weight-loss medications—were added after the initial distribution in response to increasing clinical interest in pharmacologic therapies, such as GLP-1 receptor agonists. Survey responses were collected and reported as percentages, (Supplementary Table 1; Supplementary Table 2, http://links.lww.com/JG9/A491, http://links.lww.com/JG9/A492), with graphical representations included where appropriate. This study was reviewed by the NYU Langone Health Institutional Review Board and classified as exempt human subjects research consistent with institutional policy for anonymous survey studies of adult participants, minimal risk.

## Results

**Figure 1:**
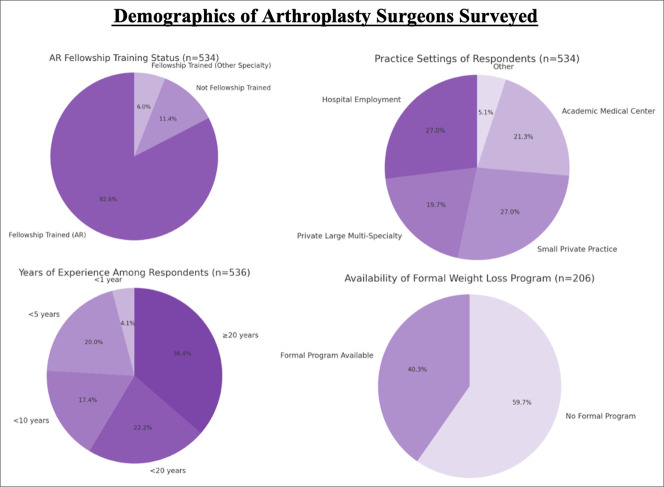
Pie chart showing demographic characteristics of arthroplasty surgeons surveyed. *Top left*: Fellowship training status (n = 534), with the darkest segment denoting fellowship trained in adult reconstruction (82.6%), intermediate shading for not fellowship trained (11.4%), and the lightest for fellowship-trained in other specialties (6.0%). *Top right*: Practice setting (n = 534), with darker segments for hospital employed (27.0%) and small private practice (27.0%), moderate shading for academic medical centers (21.3%) and large multispecialty groups (19.7%), and the lightest for other settings (5.1%). *Bottom left*: Years in practice (n = 536), with shading from darkest to lightest corresponding to ≥20 years (36.4%), <20 years (22.2%), <10 years (17.4%), <5 years (20.0%), and <1 year (4.1%). *Bottom right*: Availability of a formal weight loss program (n = 206), with lighter shading representing no program (59.7%) and darker shading for formal programs (40.3%).

Among surveyed surgeons, 82.58% (441/534) reported completing an arthroplasty reconstruction fellowship, whereas 11.42% (61/534) had no fellowship training, and 5.99% (32/534) were trained in other specialties (Figure 1).

Regarding practice setting, 26.97% (144/534) reported working in hospital employment, 26.97% (144/534) in small private practices, 21.35% (114/534) in academic medical centers, 19.66% (105/534) in large multispecialty private practices, and 5.06% (27/534) in other practice types (Supplementary Table 1, http://links.lww.com/JG9/A491).

In terms of clinical experience, 4.10% (22/536) had fewer than 1 year in practice, 19.96% (107/536) had <5 years, 17.35% (93/536) had <10 years, 22.20% (119/536) had <20 years, and 36.38% (195/536) had ≥20 years of experience (Supplementary Table 1, http://links.lww.com/JG9/A491).

When asked about institutional support, 40.29% (83/206) reported having access to a formal weight loss program at their institution, whereas 59.71% (123/206) did not (Supplementary Table 1, http://links.lww.com/JG9/A491).

**Figure 2:**
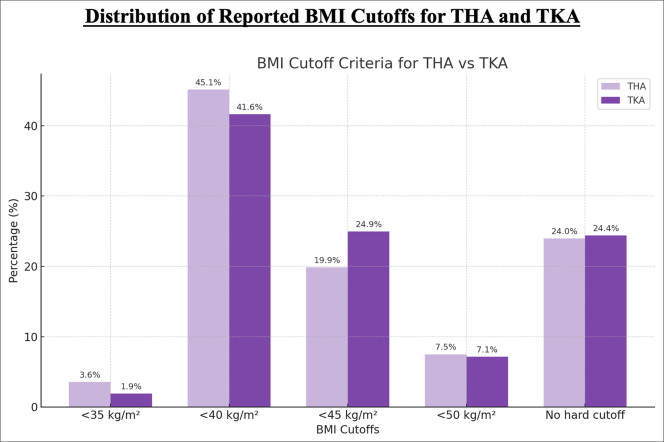
Bar chart showing distribution of reported body mass index (BMI) cutoffs for total hip arthroplasty (THA) and total knee arthroplasty (TKA). Bar chart of surgeon-reported BMI thresholds for surgical candidacy. Lavender bars represent THA and violet bars represent TKA.

Regarding BMI cutoffs and THA (Figure 2), 3.56% (19/534) of arthroplasty surgeons indicated using a BMI cutoff of less than 35 kg/m^2^, 45.13% (241/534) indicated a cutoff of less than 40 kg/m^2^, 19.85% (106/534) indicated a cutoff of less than 45 kg/m^2^, 7.49% (40/534) indicated a cutoff of less than 50 kg/m^2^, and 23.97% (128/534) indicated that there is no hard cutoff for BMI (Figure 2). In terms of BMI cutoffs and TKA, 1.88% (10/533) of arthroplasty surgeons indicated using a BMI cutoff of less than 35 kg/m^2^, 41.65% (222/533) indicated a cutoff of less than 40 kg/m^2^, 24.95% (133/533) indicated a cutoff of less than 45 kg/m^2^, 7.13% (38/533) indicated a cutoff of less than 50 kg/m^2^, and 24.39% (130/533) indicated that there is no hard cutoff for BMI (Figure 2).

**Figure 3:**
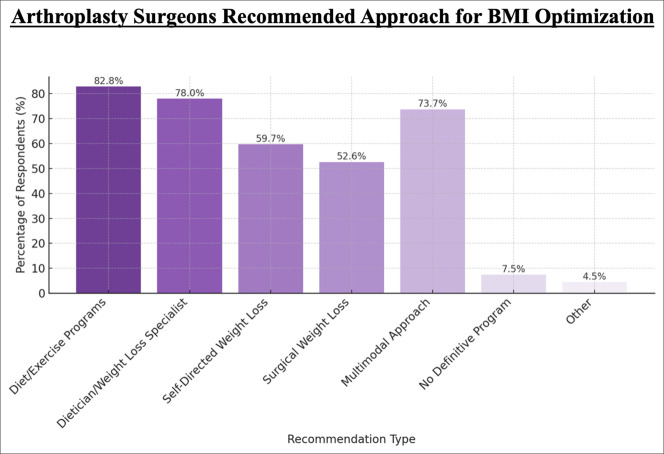
Bar chart showing recommended strategies for BMI optimization before arthroplasty. Survey responses (n = 210) reflect endorsement of weight loss interventions. Darker bars represent the most frequently recommended approaches: diet/exercise programs (82.8%) and referral to a dietician or weight loss specialist (78.0%). Intermediate and lighter bars represent self-directed weight loss (59.7%), surgical weight loss (52.6%), multimodal approach (73.7%), no definitive program (7.5%), and other strategies (4.5%).

Regarding weight optimization strategies, 82.84% (444/536) of respondents recommended structured diet and exercise programs, 77.99% (418/536) referred patients to a dietitian or weight loss specialist, 73.69% (395/536) advocated for a multimodal approach, and 59.70% (320/536) supported self-directed weight loss efforts (Figure 3). In addition, 52.61% (282/536) endorsed bariatric surgery as an option, 7.46% (40/536) reported having no structured weight loss protocol, and 4.48% (24/536) selected “other.”

**Figure 4:**
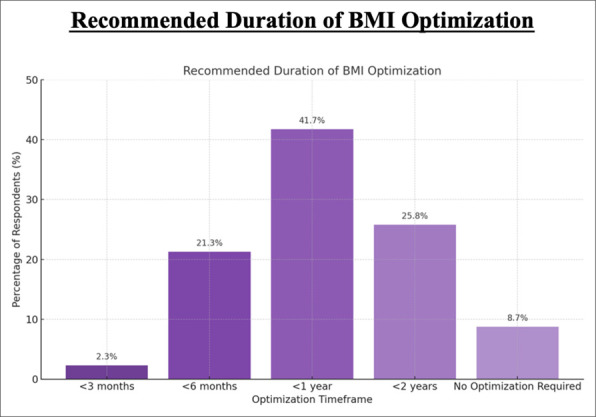
Bar chart showing recommended duration of preoperative BMI optimization. Bar graph displaying preferred optimization timeframes (n = 210) and percentage of respondents. The darkest bar corresponds to <1 year (41.7%), followed by < 2 years (25.8%), <6 months (21.3%), no optimization required (8.7%), and <3 months (2.3%), shown in progressively lighter shades.

For the duration of BMI optimization to facilitate preoperative weight loss, 2.28% (12/527) of respondents recommended at least 3 months, 21.29% (112/527) recommended at least 6 months, 41.83% (220/527) recommended at least 1 year, 25.86% (136/527) recommended at least 2 years, and 8.75% (46/527) indicated that no BMI optimization is required (Figure 4).

**Figure 5:**
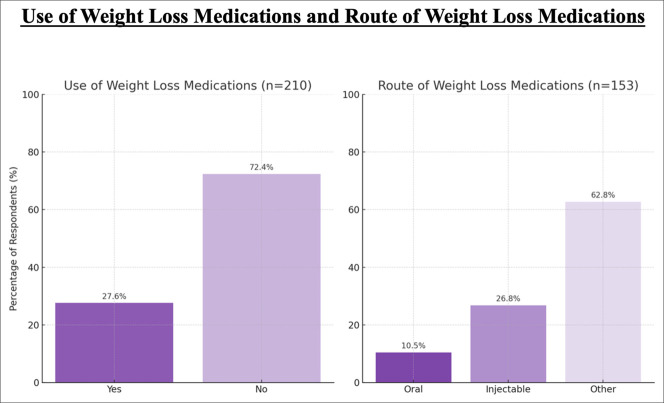
Bar chart showing use and route of weight loss medications. Left panel (n = 210): Survey responses on the use of pharmacologic weight loss agents. The darker bar indicates respondents who recommend medications (27.6%); the lighter bar indicates those who do not (72.4%). Right panel (n = 153): Reported routes of administration among those who recommend weight loss medications: darkest bar indicates oral (10.5%), intermediate bar indicates injectable (26.8%), and lightest bar indicates other or unspecified (62.8%).

Regarding the use of weight loss medications to optimize their patients, 27.62% (58/210) of arthroplasty surgeons reported their patients' using medications, such as GLP-1 agonists, lipase inhibitors, or appetite suppressants, whereas 72.38% (152/210) did not. The route of administration for weight loss medications was selected as “other” in 62.75% (n = 96/153) of respondents, followed by injectable therapies at 26.80% (n = 41/153), and oral medications at 10.46% (n = 16/153). Regarding the management of patients' weight loss medications, arthroplasty surgeons indicated that 1.01% (2/199) manage the medications themselves, whereas 98.99% (197/199) do not (Figure 5).

## Discussion

This study presented the current practices of arthroplasty surgeons in managing patients with obesity undergoing TJA. In comparison to an AAHKS survey conducted in 2021(n = 675 participants), our survey had a similar distribution of BMI cutoffs for THA and TKA.^16^ However, our survey demonstrated that 24% (128/534) of surgeons reported not using strict BMI cutoffs, whereas the 2021 AAHKS survey demonstrated that 13% (84/675) used no strict cutoff.^16^ Although these two surveys represent different cohorts, the higher proportion of surgeons reporting no strict BMI cutoff (24% versus 13%) describes variability in current approaches to BMI optimization among arthroplasty surgeons. For example, 28% (58/210) of respondents reported that their patients are using weight loss medications. In addition, most respondents favored diet and exercise-based weight loss programs (83%, n = 444/534) and weight loss specialist referral (78%, n = 418/534), which demonstrates that current arthroplasty surgeons are facilitating a multimodal approach to BMI optimization for TJA patients with obesity.

In addition, our survey revealed that arthroplasty surgeons are factoring in the timing of BMI reductions before TJA. For example, 68% (356/526) of respondents reported allowing up to one or 2 years for BMI optimization before TJA. This preference for timing demonstrates that most surgeons do not advocate for rapid weight loss occurring in less than 9 months, which has been associated with an increased risk of PJI in TKA patients with morbid obesity and increased PJI and surgical site infection in THA patients with morbid obesity.^18,19^

In comparing approaches to managing patients with elevated BMI undergoing TJA, the 2021 AAHKS survey found that 72.4% of respondents suggested bariatric surgery either always or sometimes.^16^ By contrast, our survey revealed that bariatric surgery was advocated by only 52.6% (282/536) of respondents. Although these data reflect two different surveyed cohorts, there is variability between the recommendations for bariatric surgery before TJA (72.4% versus 52.6%). This may reflect the mixed results of bariatric surgery in the literature^20,21^ and the current availability of alternative approaches, such as GLP-1 agonists.

To our knowledge, this is the first survey to specifically quantify the extent to which arthroplasty surgeons consider pharmacologic agents, including GLP-1 agonists, for preoperative BMI reduction. In our cohort, 28% (58/210) of respondents indicated that their patients used weight loss medications to support weight reduction; however, only 1% (2/199) of surgeons reported personally managing these therapies. These findings suggest that surgeons are largely relying on a multidisciplinary approach to weight management. Nearly 40% (83/206) of respondents reported access to a formal weight loss program at their home institution, and a substantial majority (77.99%, n = 418/536) frequently referred patients to dietitians or weight loss specialists to support BMI optimization.

Given that arthroplasty surgeons predominantly favored exercise and nutritional strategies for BMI optimization, although only a minority managed pharmacological treatments, there is an urgent need to evaluate the effectiveness of multidisciplinary team–based interventions. For example, access to institutional weight loss programs was limited, with fewer than half of respondents indicating availability at their facilities. This observation reflects a broader lack of standardization in weight management pathways and emphasizes the need for prospective studies to define best practices. Studies investigating the value of referrals to nutritionists, physical therapists, and pharmacological weight loss specialists for preoperative TJA patients with obesity could help standardize care, providing arthroplasty surgeons with an optimal multidisciplinary approach to improving metabolic health before TJA.

## Limitations

One limitation of our study is the potential for selection bias, as the survey was distributed through AAHKS mailing lists and social media platforms, potentially excluding relevant respondents. In addition, the self-reported nature of survey responses introduces the possibility of recall bias or inaccuracies in reporting. Geographic identifiers were not collected, limiting evaluation of regional heterogeneity in reported practices. Importantly, the survey did not assess the rationale behind surgeons' decisions to recommend or avoid specific weight loss interventions, limiting our understanding of their clinical preferences. Finally, because questions regarding pharmacologic weight loss interventions were added after initial distribution, the response rate for these items were lower.

## Conclusion

Our national survey reveals that most sampled arthroplasty surgeons recommend a multidisciplinary approach that incorporates referrals to weight loss specialists, nutritionists, and primary care providers to support preoperative optimization. Although a minority of arthroplasty surgeons directly manage the prescriptions of weight loss medications, our data reveal that nearly one-quarter of arthroplasty surgeons report that their patients are being treated with weight loss promoting medications, highlighting a trend toward integrating pharmacologic strategies into preoperative care.

## Supplementary Material
