# Supplementary material for: Practice Patterns in Body Mass Index Optimization Among US Arthroplasty Surgeons: Results of a National American Association of Hip and Knee Surgeons Survey
Source: J Am Acad Orthop Surg Glob Res Rev. 2026 Feb 17;10(2):e25.00187. doi: 10.5435/JAAOSGlobal-D-25-00187 (PMC12915733; doi:10.5435/JAAOSGlobal-D-25-00187)
Supplement: Supplementary file 1 [file jagrr-10-e25.00187-s001.docx]

**Table 1: Demographics of Survey Respondents**

| **Question** | **Answer Choices** | **Response Percent** | **Responses** |
| --- | --- | --- | --- |
| Q1. Are you AR fellowship trained? | Yes | 82.58% | 441 |
|  | No | 11.42% | 61 |
|  | Fellowship trained in other specialty | 5.99% | 32 |
|  | **Total Answered:** |  | 534 |
| Q2. In what setting do you practice? | Hospital employment | 26.97% | 144 |
|  | Private large multi-specialty practice/organization | 19.66% | 105 |
|  | Small private practice | 26.97% | 144 |
|  | Academic university medical center | 21.35% | 114 |
|  | Other | 5.06% | 27 |
|  | **Total Answered:** |  | 534 |
| Q3. How long have you been in practice? | <1 year | 4.10% | 22 |
|  | <5 years | 19.96% | 107 |
|  | <10 years | 17.35% | 93 |
|  | <20 years | 22.20% | 119 |
|  | ≥20 years | 36.38% | 195 |
|  | **Total Answered:** |  | 536 |
| Q4. Do you have a formal weight loss program at your institution? | Yes | 40.29% | 83 |
|  | No | 59.71% | 123 |
|  | **Total Answered:** |  | **206** |
