# Supplementary material for: Practice Patterns in Body Mass Index Optimization Among US Arthroplasty Surgeons: Results of a National American Association of Hip and Knee Surgeons Survey
Source: J Am Acad Orthop Surg Glob Res Rev. 2026 Feb 17;10(2):e25.00187. doi: 10.5435/JAAOSGlobal-D-25-00187 (PMC12915733; doi:10.5435/JAAOSGlobal-D-25-00187)
Supplement: Supplementary file 2 [file jagrr-10-e25.00187-s002.docx]

| **Table 2: Pre-Operative Period Protocols for High BMI Patients**   \| **Question** \| **Answer Choices** \| **Response Percent** \| **Responses** \| \| --- \| --- \| --- \| --- \| \| Q5. What is your BMI cutoff for patients undergoing THA? \| BMI <35kg/m2 \| 3.56% \| 19 \| \|  \| BMI <40kg/m2 \| 45.13% \| 241 \| \|  \| BMI <45kg/m2 \| 19.85% \| 106 \| \|  \| BMI <50kg/m2 \| 7.49% \| 40 \| \|  \| No hard cutoff \| 23.97% \| 128 \| \|  \| **Total Answered** \|  \| **534** \| \| Q6. What is your BMI cutoff for patients undergoing TKA? \| BMI <35kg/m2 \| 1.88% \| 10 \| \|  \| BMI <40kg/m2 \| 41.65% \| 222 \| \|  \| BMI <45kg/m2 \| 24.95% \| 133 \| \|  \| BMI <50kg/m2 \| 7.13% \| 38 \| \|  \| No hard cutoff \| 24.39% \| 130 \| \|  \| **Total Answered** \|  \| **533** \| \| Q7. What do you recommend to patients in order to optimize their BMI? (Include all that apply) \| Weight loss programs: diet/exercise structured \| 82.84% \| 444 \| \|  \| Dietician referral/weight loss specialist \| 77.99% \| 418 \| \|  \| Support patient self-directed weight loss regimen \| 59.7% \| 320 \| \|  \| Surgical weight loss \| 52.61% \| 282 \| \|  \| Multimodal approach \| 73.69% \| 395 \| \|  \| No definitive program \| 7.46% \| 40 \| \|  \| Other \| 4.48% \| 24 \| \|  \| **Total Answered** \|  \| **536** \| \| Q8. How often do you have patient's follow up who are undergoing BMI optimization prior to their joint replacement? \| <1 month \| 3.93% \| 21 \| \|  \| 3 months \| 61.68% \| 330 \| \|  \| 6 months \| 14.02% \| 75 \| \|  \| 1 year \| 0.56% \| 3 \| \|  \| PRN \| 19.81% \| 106 \| \|  \| **Total Answered** \|  \| **535** \| \| Q9. How long before you perform a joint replacement on a patient who underwent surgical weight loss? \| <1 month \| 2.25% \| 12 \| \|  \| 3 months \| 19.51% \| 104 \| \|  \| 6 months \| 42.4% \| 226 \| \|  \| 1 year \| 22.51% \| 120 \| \|  \| >1 year \| 3.38% \| 18 \| \|  \| Other \| 9.94% \| 53 \| \|  \| **Total Answered** \|  \| **533** \| \| Q10. For patients whom you are optimizing weight what is your criteria for proceeding with surgery? \| BMI cutoff \| 45.9% \| 246 \| \|  \| Weight loss cutoff/weight loss number \| 20.34% \| 109 \| \|  \| Percentage cutoff \| 7.28% \| 39 \| \|  \| No cutoff \| 20.71% \| 111 \| \|  \| Other \| 5.78% \| 31 \| \|  \| **Total Answered** \|  \| **536** \| \| Q11. What is the duration of BMI Optimization for Patients Undergoing Weight Loss? \| <3 months \| 2.28% \| 12 \| \|  \| <6 months \| 21.29% \| 112 \| \|  \| <1 year \| 41.83% \| 220 \| \|  \| <2 years \| 25.86% \| 136 \| \|  \| No BMI optimization required \| 8.75% \| 46 \| \|  \| **Total Answered** \| **100%** \| **526** \| \| Q12. Do you make any exceptions to patients above your operative BMI criteria? \| Age \| 3.45% \| 17 \| \|  \| Low Charlson Comorbidity index (CCI) \| 19.47% \| 96 \| \|  \| Exercise tolerance \| 4.06% \| 20 \| \|  \| Socioeconomic status \| 1.01% \| 5 \| \|  \| No BMI optimization required \| 18.46% \| 91 \| \|  \| Other \| 53.55% \| 264 \| \|  \| **Total Answered** \|  \| **493** \| \| Q13. Have you used weight loss medication to optimize your patients? (i.e. GLP-1 agonist, lipase inhibitor, appetite suppressant, etc.) \| Yes \| 27.62% \| 58 \| \|  \| No \| 72.38% \| 152 \| \|  \| **Total Answered** \|  \| **210** \| \| Q14. What route of weight loss medication did you use to optimize your patients? \| Oral \| 10.46% \| 16 \| \|  \| Injectable \| 26.8% \| 41 \| \|  \| Other \| 62.75% \| 96 \| \|  \| **Total Answered** \|  \| **153** \| \| Q15. Do you manage your patient's weight loss medications, if used? \| Yes \| 1.01% \| 2 \| \|  \| No \| 98.99% \| 197 \| \|  \| **Total Answered** \|  \| **199** \| \| Q16. How long did you wait after weight loss medication administration before you performed your THA/TKA? \| <1 week \| 5.56% \| 9 \| \|  \| <2 weeks \| 24.69% \| 40 \| \|  \| <4 weeks \| 5.56% \| 9 \| \|  \| <3 months \| 17.9% \| 29 \| \|  \| <6 months \| 22.84% \| 37 \| \|  \| <1 year \| 15.43% \| 25 \| \|  \| >/= 1 year \| 8.02% \| 13 \| \|  \| **Total Answered** \|  \| **162** \| |
| --- | --- | --- | --- | --- | --- | --- | --- | --- | --- | --- | --- | --- | --- | --- | --- | --- | --- | --- | --- | --- | --- | --- | --- | --- | --- | --- | --- | --- | --- | --- | --- | --- | --- | --- | --- | --- | --- | --- | --- | --- | --- | --- | --- | --- | --- | --- | --- | --- | --- | --- | --- | --- | --- | --- | --- | --- | --- | --- | --- | --- | --- | --- | --- | --- | --- | --- | --- | --- | --- | --- | --- | --- | --- | --- | --- | --- | --- | --- | --- | --- | --- | --- | --- | --- | --- | --- | --- | --- | --- | --- | --- | --- | --- | --- | --- | --- | --- | --- | --- | --- | --- | --- | --- | --- | --- | --- | --- | --- | --- | --- | --- | --- | --- | --- | --- | --- | --- | --- | --- | --- | --- | --- | --- | --- | --- | --- | --- | --- | --- | --- | --- | --- | --- | --- | --- | --- | --- | --- | --- | --- | --- | --- | --- | --- | --- | --- | --- | --- | --- | --- | --- | --- | --- | --- | --- | --- | --- | --- | --- | --- | --- | --- | --- | --- | --- | --- | --- | --- | --- | --- | --- | --- | --- | --- | --- | --- | --- | --- | --- | --- | --- | --- | --- | --- | --- | --- | --- | --- | --- | --- | --- | --- | --- | --- | --- | --- | --- | --- | --- | --- | --- | --- | --- | --- | --- | --- | --- | --- | --- | --- | --- | --- | --- | --- | --- | --- | --- | --- | --- | --- | --- | --- | --- | --- | --- | --- | --- | --- | --- | --- | --- | --- | --- | --- | --- | --- | --- | --- | --- | --- | --- | --- | --- | --- | --- | --- | --- | --- | --- | --- | --- | --- | --- | --- | --- | --- | --- | --- | --- | --- | --- | --- | --- | --- | --- | --- | --- | --- | --- | --- | --- | --- | --- | --- | --- | --- | --- | --- | --- | --- | --- | --- | --- | --- |
